# Monitoring Mosquito-Borne Arbovirus in Various Insect Regions in China in 2018

**DOI:** 10.3389/fcimb.2021.640993

**Published:** 2021-03-11

**Authors:** Yuan Fang, Wei Zhang, Jing-Bo Xue, Yi Zhang

**Affiliations:** ^1^ National Institute of Parasitic Diseases, Chinese Center for Disease Control and Prevention, Shanghai, China; ^2^ Chinese Center for Tropical Diseases Research, Ministry of Science and Technology, Shanghai, China; ^3^ Key Laboratory of Parasite and Vector Biology, Ministry of Health, Shanghai, China; ^4^ WHO Collaborating Centre for Tropical Diseases, Shanghai, China; ^5^ National Center for International Research on Tropical Diseases, Ministry of Science and Technology, Shanghai, China; ^6^ Zichuan District Center for Disease Control and Prevention, Zibo, China

**Keywords:** Japanese encephalitis virus, Getah virus, insect-specific flavivirus, *Culex*, *Anopheles*

## Abstract

**Background:**

Increases in global travel and trade are changing arbovirus distributions worldwide. Arboviruses can be introduced by travelers, migratory birds, or vectors transported *via* international trade. Arbovirus surveillance in field-collected mosquitoes may provide early evidence for mosquito-borne disease transmission.

**Methods:**

During the seasons of high mosquito activity of 2018, 29,285 mosquitoes were sampled from seven sentinel sites in various insect regions. The mosquitoes were analyzed by RT-PCR for alphaviruses, flaviviruses, and orthobunyaviruses.

**Results:**

We detected three strains of Japanese encephalitis virus (JEV), five strains of Getah virus (GETV), and 45 strains of insect-specific flaviviruses including Aedes flavivirus (AeFV, 1), Chaoyang virus (CHAOV, 1), Culex flavivirus (CxFV, 17), Hanko virus (HANKV, 2), QuangBinh virus (QBV, 22), and Yunnan Culex flavivirus (YNCxFV, 2). Whole genomes of one strain each of GETV, CxFV, CHAOV, and AeFV were successfully amplified. Phylogenetic analysis revealed that the new JEV strains detected in the Shanghai and Hubei Provinces belong to the GI-b strain and are phylogenetically close to the NX1889 strain (MT134112) isolated from a patient during a JE outbreak in Ningxia in 2018. GETVs were found in Inner Mongolia, Hubei, and Hainan and belonged to Group III. They were closely related to strains isolated from swine. HANKV was recorded for the first time in China and other ISFVs were newly detected at several sentinel sites. The bias-corrected maximum likelihood estimation value for JEV in Jinshan, Shanghai was 4.52/1,000 (range 0.80–14.64). Hence, there is a potential risk of a JEV epidemic in that region.

**Conclusion:**

GI-b is the dominant circulating JEV genotype in nature and poses a health risk to animals and humans. The potential threat of widespread GETV distribution as a zoonosis is gradually increasing. The present study also disclosed the dispersion and host range of ISFVs. These findings highlight the importance of tracing the movements of the vectors and hosts of mosquito-borne pathogens in order to prevent and control arbovirus outbreaks in China.

## Introduction

Mosquito-borne diseases are prevalent worldwide. Emergent and established tropical diseases are spreading faster than expected. Dengue outbreaks have occurred in China over the last decade. These occurred in Yunnan [2013, (Zhang et al., 2014)], Guangdong [2014, (Xiao et al., 2016)], Fujian [2016, (Han et al., 2018)], and Zhejiang [2017, (Yan et al., 2018)] and of them emerged in heretofore dengue-free or low endemic areas. The number and spatial distribution of dengue cases in China in 2019 reached an unprecedented level. Morbidity was 1.63/100,000 and was second in severity only to that of the major dengue outbreak of 2014 (Liu, 2020). Summer monsoons and climate change have substantial impacts on dengue control and prevention in southeastern China (Liu et al., 2020a). Japanese encephalitis (JE) has been effectively controlled by vaccination. However, JE outbreaks have been recorded in adults in Shanxi [2006, (Wang et al., 2007)], Hubei [2009–2010, (Hu et al., 2013)], Shandong [2013 (Tao et al., 2014; Li et al., 2019)], Gansu [2018, (Tian and Yang, 2019)], and Ningxia [2018, (Liu et al., 2020b)]. The potential resurgence of malaria (Feng et al., 2020) and the emergence of Zika (Song et al., 2017), Chikungunya (Wu et al., 2012), and West Nile (Cao et al., 2019) virus threaten public health.

Before disease outbreaks occur, viruses colonize in field mosquitoes and circulate in nature. Mosquito-borne disease outbreaks reflect inadequate mosquito-borne pathogen surveillance. Documenting the dynamics of pathogen-bearing mosquitoes in the field might herald imminent mosquito-borne disease outbreaks (Masetti et al., 2008). Sporadic mosquito pathogen detection has been conducted in China to a limited extent. In addition, baseline surveys have not yet been performed in certain areas. Hence, surveys are needed to determine the geographic distribution of arboviruses in various regions of China. The mosquito surveillance system in China has focused mainly on vector diversity and abundance (Wu et al., 2017; Guo et al., 2019). To the best of our knowledge, however, only a few districts have sustained longitudinal mosquito-borne pathogen surveillance. Moreover, large-scale latitudinal surveillance is uncommon. In 2018, we established seven sentinel sites in China based on ecological niches for mosquito-borne pathogen surveillance. Alphaviruses, flaviviruses, and orthobunyaviruses were detected and their endemic risks were evaluated in target areas.

## Methods

### Mosquito Sampling

**Figure 1:**
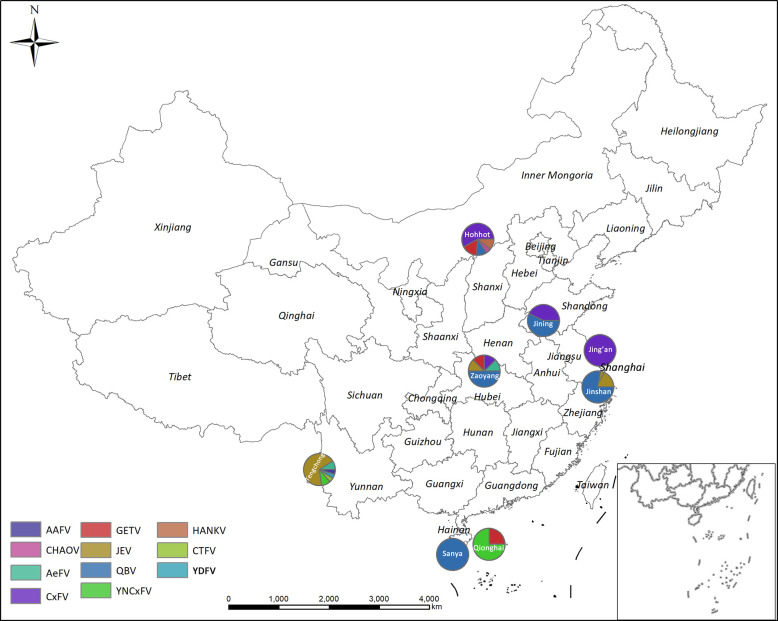
Arbovirus distributions by mosquito pathogen surveillance sentinel site in China in 2018. AAFV, Anopheles-associated flavivirus; AeFV, Aedes flavivirus; CHAOV, Chaoyang virus; CTFV, Culex theileri flavivirus; CxFV, Culex flavivirus; HANKV, Hanko virus; JEV, Japanese encephalitis virus; QBV, Quang Binh virus; YDFV, Yamadai flavivirus; YNCxFV, Yunnan Culex flavivirus.

Sentinel sites were established in various insect regions (IR) of China according to geographical insect division (Shen et al., 2013). The areas included Jining, Shandong Province (Northern China IR), Hohhot, Inner Mongolia Autonomous Region (Northeastern China IR), Jin’an and Jinshan Districts, Shanghai Municipality (Eastern China), Sanya City and Qionghai County in Hainan Province (Southern China Hainan sub-IR), and Zaoyang County in Hubei Province (Changjiang-Huaihe IR). **Figure 1** is a sentinel site map generated by ArcGIS v. 10.1 ArcMap software (ESRI, Redlands, CA, USA). Mosquitoes were collected from July to October 2018 using UV light traps (Kungfu Dude Mosquito & Fly Trap, Wuhan Ji Xing Medical Technology Co., Wuhan, China) and the labor hour method (Fang et al., 2019). The collection sites covered various ecological characteristics and included residential areas, gardens, office workplaces, schools, and livestock. Both sampling methods were performed twice monthly at 15-day intervals. Aedine larvae and pupae were collected from positive containers during a Breteau Index survey, brought to a laboratory, and reared until adult emergence. Mosquitoes were identified according to a national key using morphological characteristics (Lu, 1997). Ambiguous specimens were confirmed by molecular methods (Fang et al., 2017). Mosquitoes were pooled by species and collection date, method, and location and stored at -80°C until further testing. There was a maximum of 100 individuals per pool.

### Nucleic Acid Extraction and Polymerase Chain Reaction

RNA extraction, cDNA synthesis, and RNA integrity assessment were performed as previously described (Fang et al., 2018). PCR amplification to detect alphaviruses, flaviviruses, and orthobunyaviruses was performed as described by Fang et al. (2021a). For additional genotype identification, the primer sets JEV-Ef/JEV-Er (Gao et al., 2013) and CxFV-E-F/CxFV-E-R (Saiyasombat et al., 2010) amplified the *E* genes of JEV and CxFV, respectively. The *E2* gene of the Getah virus (GETV) was amplified by the primer sets GETVE2F/GETVE2R (Zhai et al., 2008). The PCR products were visualized on 1% or 2% agarose gel in 0.5× Tris-acetate-EDTA buffer with Goldview. Positive products were purified, cloned and sequenced by Sangon (Shanghai, China).

### Whole-Genome Sequencing

Primer Premier v. 5.0 (Premier Biosoft International, Palo Alto, CA, USA) was used to analyze the molecular characteristics and putative pathogenesis mechanisms of the viruses. It designed primers to amplify the complete genomes of the Getah (GETV), Chaoyang (CHAOV), and Quang Binh (QBV) viruses and Culex flavivirus (CxFV) using the local SC201807 (MK693225), HLD15 (NC_017086), JM17156/China/2017 (MH827524), and DG1064 (JQ308188) isolates, respectively, as references. The PCR products were sent for Sanger sequencing and applied towards the design of the subsequent primers.

### Phylogenetic Analysis

PCR product sequences were compared with those deposited in the GenBank database using the BLAST program. Multiple sequence alignments were generated with ClustalW2 using fragments of the homologous NS5, JEV E, and GETV E2 genes available in GenBank and the sequences obtained in this study. Default ClustalW2 settings were manually adjusted as required (Larkin et al., 2007). Neighbor-joining (NJ) trees were plotted following Kimura’s two-parameter (K2P) distance model (Kimura, 1980) and 1,000 bootstrap replicates in MEGA v. 7.0 (Kumar et al., 2016). Based on the Akaike Information Criterion (AIC), a best-fit alignment model was determined with Modeltest v. 3.7 and PAUP* v. 4.0b10 (Wilgenbusch and Swofford, 2003). The maximum likelihood (ML) and Bayesian likelihood trees were plotted using the GTR+I+G model for flavivirus *NS5* and the Japanese encephalitis virus (JEV) *E* genes. The ML and Bayesian likelihood trees were plotted using the GTR+G model for the GETV *E2* and the Culex flavivirus (CxFV E) genes. The ML tree was plotted with MEGA v. 7.0 using 1,000 bootstrap replicates. The Bayesian tree was plotted with MrBayes v. 3.2.1 (Ronquist et al., 2012) and run for 10 million generations of which 25% were discarded as burn-in. The trees were unrooted for the least biased topology and visualized in Figtree v. 1.4.2 (http://tree.bio.ed.ac.uk/software/figtree/).

### Infection Rate Calculation

Mosquito pool sizes varied considerably. Therefore, infection rates were calculated using a bias-corrected maximum likelihood estimation (MLE) and a minimum infection rate (MIR) in the Excel add-in PooledInfRate v. 4 statistical software package (Biggerstaff, 2006). Rates were expressed as the number of infected mosquitoes per 1,000 collected.

## Results

### Detection of Mosquito-Borne Pathogens From Samples

A total of 29,285 mosquitoes including *Anopheles sinensis*, *Culex* spp., *Aedes albopictus*, and *Armigeres subalbatus* were collected at the seven sentinel sites during the mosquito activity season of 2018. All samples in 870 pools were tested for the presence of mosquito-borne pathogens. Collection data for each sentinel site are listed in **Table S1**. *Cx.* spp. including *Cx. pipiens*, *Cx. tritaeniorhychus*, and *Cx. quinquefasciatus* predominated (27,949 in 623 pools) and accounted for 95.44% of the total. They were followed by *An. sinensis* (1.66%, 486/47 pools), *Ae. albopictus* (2.43%, 713/183 pools), and *Ar. subalbatus* (0.47%, 137/17 pools). Intact RNA was successfully extracted from all mosquito pools and confirmed by 18S rRNA amplification (Hoffmann et al., 2004). Forty-eight flavivirus and five GETV strains were recovered by successful amplification of the partial flavivirus *NS5* and the alphavirus *NSP1* gene, respectively. The 48 flavivirus strains comprised one Aedes flavivirus (AeFV), one Chaoyang virus (CHAOV), 16 Culex flavivirus (CxFV), three JEV, two Hankovirus (HANKV), 22 Quang Binh virus (QBV), and three Yunnan Culex flavivirus (YNCxFV) strains. The *E* genes of JEV and CxFV were successfully amplified in three and nine positive pools, respectively. Four positive amplification and sequence determination results were obtained for the GETV *E2* gene. No orthobunyaviral RNA sequences were detected. The species names, collection information, host species, and GenBank accession numbers of the arbovirus strains obtained here are shown in **Table 1**.

**Table 1:** Mosquito-borne viruses detected at different sentinel sites during the mosquito activity seasons of 2018.

Strain	Virus	Host	Collection date	Geographic location	GenBank ID		
					*NS5*	*E*	Whole genome
HB_C9_18-7-HZ-Aea-B-4-JG-1	AeFV	*Aedes albopictus*	7-Jul-2018	China: Zaoyang, Hubei Province	MW246700		
NM,JA_F7_18-8L-NH-Cxp-Y-2-1	CHAOV	*Culex pipiens*	25-Aug-2018	China: Hohhot, Inner Mongolia	MW246701		MW246770
HB_F3_18-8E-HZ-C-Y-5-1	CxFV	*Cx. tritaeniorhynchus*	13-Aug-2018	China: Zaoyang, Hubei Province	MW246702		
JN,SY,TC_F3_18-8L-S-J-Cxt-Y-4-1		*Cx. tritaeniorhynchus*	24-Aug-2018	China: Jining, Shandong Province	MW246703	MW246760	
JS,JA_A4_18-9E-SJ-Cxp-Y-1-1		*Cx. pipiens*	10-Sep-2018	China: Jining, Shandong Province	MW246704	MW246767	MW246772
JS,JA_A9_18-9L-SJ-Cxp-Y-3-1		*Cx. pipiens*	26-Sep-2018	China: Jining, Shandong Province	MW246705	MW246768	
JS,JA_D9_18-10M-JA-Cxp-C-4		*Cx. pipiens*	11-Oct-2018	China: Jin’an, Shanghai Municipality	MW246706		
NM,JA_A3_18-9M-NH-Cxp-Y-1-1		*Cx. pipiens*	14-Sep-2018	China: Hohhot, Inner Mongolia	MW246707		
NM,JA_C3_18-8M-NH-Cxp-Y-1-1		*Cx. pipiens*	14-Aug-2018	China: Hohhot, Inner Mongolia	MW246708	MW246761	
NM,JA_C5_18-8E-NH-Cxp-Y-2-1		*Cx. pipiens*	10-Aug-2018	China: Hohhot, Inner Mongolia	MW246709		
NM,JA_C6_18-7L-NH-Cxp-Y-1-1		*Cx. pipiens*	25-Jul-2018	China: Hohhot, Inner Mongolia	MW246710		
NM,JA_D5_18-7L-NH-Cxp-Y-2-1		*Cx. pipiens*	25-Jul-2018	China: Hohhot, Inner Mongolia	MW246711		
NM,JA_D7_18-8M-NH-Cxp-Y-1-1		*Cx. pipiens*	14-Aug-2018	China: Hohhot, Inner Mongolia	MW246712		
NM,JA_E11_18-8M-NH-Cxp-Y-3-1		*Cx. pipiens*	14-Aug-2018	China: Hohhot, Inner Mongolia	MW246713	MW246762	
NM,JA_E12_18-8M-NH-Cxp-Y-3-1		*Cx. pipiens*	14-Aug-2018	China: Hohhot, Inner Mongolia	MW246714	MW246763	
NM,JA_E7_18-8M-NH-Cxp-Y-2-3		*Cx. pipiens*	14-Aug-2018	China: Hohhot, Inner Mongolia	MW246715	MW246764	
NM,JA_G2_18-8L-NH-Cxp-Y-3		*Cx. pipiens*	25-Jul-2018	China: Hohhot, Inner Mongolia	MW246716	MW246765	
NM,JA_G3_18-8L-NH-Cxp-Y-2-4		*Cx. pipiens*	25-Jul-2018	China: Hohhot, Inner Mongolia	MW246717	MW246766	
NM,JA_E12_18-8M-NH-Cxp-Y-3-1	GETV	*Cx. pipiens*	14-Aug-2018	China: Hohhot, Inner Mongolia	MW246718	MW246755	
NM,JA_F2_18-8L-NH-Cxp-Y-1-1		*Cx. pipiens*	25-Jul-2018	China: Hohhot, Inner Mongolia	MW246719	MW246753	MW246769
NM,JA_G4_18-8L-NH-Cxp-Y-2-4		*Cx. pipiens*	25-Jul-2018	China: Hohhot, Inner Mongolia	MW246720	MW246756	
HB_A3_18-7E-HZ-ANS-Y-1-1		*Anopheles sinensis*	16-Jul-2018	China: Zaoyang, Hubei Province	MW246721	MW246754	
JS,QH_G7_18-7L-QH-Cxt-5-4		*Cx. tritaeniorhynchus*	24-Jul-2018	China: Qionghai city, Hainan Province	MW246722		
HB_B4_18-7E-HZ-C-Y-5-4	JEV	*Cx. tritaeniorhynchus*	19-Jul-2018	China: Zaoyang, Hubei Province	MW246723	MW246759	
JS,QH_B4_18-7E-JS-Cxt-C-9-6		*Cx. tritaeniorhynchus*	4-Jul-2018	China: Jinshan, Shanghai Municipality	MW246724	MW246757	
JS,QH_D3_18-8E-JS-Cxt-C-8-4		*Cx. tritaeniorhynchus*	7-Aug-2018	China: Jinshan, Shanghai Municipality	MW246725	MW246758	
NM,JA_C7_18-7L-NH-Cxp-Y-1-1	HANKV	*Cx. pipiens*	25-Jul-2018	China: Hohhot, Inner Mongolia	MW246726		
NM,JA_E2_18-8M-NH-Cxp-Y-2-1		*Cx. pipiens*	14-Aug-2018	China: Hohhot, Inner Mongolia	MW246727		
JS,JA_A5_18-9E-SJ-Cxp-Y-2-1	QBV	*Cx. pipiens*	10-Sep-2018	China: Jining, Shandong Province	MW246728		
JS,JA_A7_18-9L-SJ-Cxp-Y-1-1		*Cx. pipiens*	26-Sep-2018	China: Jining, Shandong Province	MW246729		
JS,JA_C12_18-10E-SJ-Cxp-3-1		*Cx. pipiens*	10-Oct-2018	China: Jining, Shandong Province	MW246730		
JN,SY,TC_E2_18-8E-S-J-Cxp-Y-4-1		*Cx. tritaeniorhynchus*	6-Aug-2018	China: Jining, Shandong Province	MW246731		
HB_E10_18-8E-HZ-A-Y-2-1		*An. sinensis*	13-Aug-2018	China: Zaoyang, Hubei Province	MW246732		
HB_E2_18-7L-HZ-C-S-1		*Cx. tritaeniorhynchus*	24-Jul-2018	China: Zaoyang, Hubei Province	MW246733		
HB_E5_18-8E-HZ-C-Y-1-1		*Cx. tritaeniorhynchus*	13-Aug-2018	China: Zaoyang, Hubei Province	MW246734		
HB_E7_18-8E-HZ-A-Y-1-1		*An. sinensis*	13-Aug-2018	China: Zaoyang, Hubei Province	MW246735		
JN,SY,TC_B5_18-7E-H-S-Cxt-Y-5		*Cx. tritaeniorhynchus*	10-Jul-2018	China: Sanya, Hainan Province	MW246736		
JN,SY,TC_B6_18-7E-H-S-Cxt-Y-5		*Cx. tritaeniorhynchus*	10-Jul-2018	China: Sanya, Hainan Province	MW246737		
JN,SY,TC_B7_18-7E-H-S-Cxt-Y-5		*Cx. tritaeniorhynchus*	10-Jul-2018	China: Sanya, Hainan Province	MW246738		
JN,SY,TC_B8_18-7E-H-S-Cxt-Y-5		*Cx. tritaeniorhynchus*	10-Jul-2018	China: Sanya, Hainan Province	MW246739		
JN,SY,TC_D1_18-7L-H-S-Cxt-Y-5		*Cx. tritaeniorhynchus*	10-Jul-2018	China: Sanya, Hainan Province	MW246740		
JS,JA_H6_18-10E-JS-Cxt-C-2-1		*Cx. tritaeniorhynchus*	8-Oct-2018	China: Jinshan, Shanghai Municipality	MW246741		MW246771
JS,QH_A7_18-7E-JS-Cxt-C-8-5		*Cx. tritaeniorhynchus*	4-Jul-2018	China: Jinshan, Shanghai Municipality	MW246742		
JS,QH_B5_18-7E-JS-Cxt-C-9-7		*Cx. tritaeniorhynchus*	4-Jul-2018	China: Jinshan, Shanghai Municipality	MW246743		
JS,QH_B8_18-7E-JS-Cxt-C-10-1		*Cx. tritaeniorhynchus*	4-Jul-2018	China: Jinshan, Shanghai Municipality	MW246744		
JS,QH_C3_18-7M-JS-Cxt-C-8-1		*Cx. tritaeniorhynchus*	16-Jul-2018	China: Jinshan, Shanghai Municipality	MW246745		
JS,QH_D7_18-8E-JS-Cxt-C-8-8		*Cx. tritaeniorhynchus*	7-Aug-2018	China: Jinshan, Shanghai Municipality	MW246746		
JS,QH_E11_18-8L-JS-Cxt-C-3-1		*Cx. tritaeniorhynchus*	23-Aug-2018	China: Jinshan, Shanghai Municipality	MW246747		
NM,JA_C12_18-7L-NH-Cxp-Y-1-3		*Cx. pipiens*	25-Jul-2018	China: Hohhot, Inner Mongolia	MW246748		
NM,JA_D8_18-8M-NH-Cxp-Y-1-2		*Cx. pipiens*	14-Aug-2018	China: Hohhot, Inner Mongolia	MW246749		
JS,QH_F10_18-7L-H-Q-Cxt-Y-1-1	YNCxFV	*Cx. tritaeniorhynchus*	24-Jul-2018	China: Qionghai, Hainan Province	MW246750		
JS,QH_F2_18-7E-H-Q-Cxt-Y-1-1		*Cx. tritaeniorhynchus*	10-Jul-2018	China: Qionghai, Hainan Province	MW246751		
JS,QH_F5_18-7E-H-Q-Cxt-Y-5-1		*Cx. tritaeniorhynchus*	10-Jul-2018	China: Qionghai, Hainan Province	MW246752		

AeFV, Aedes flavivirus, CHAOV, Chaoyang virus; CxFV, Culex flavivirus; E, Envelope gene; GETV, Getah virus; HANKV, Hanko virus; JEV, Japanese encephalitis virus; NS5, Non-structural 5 gene, YNCxFV, Yunnan Culex flavivirus.

AeFV was present in one pool of *Ae. albopictus* from Zaoyang, Hubei Province. CHAOV was present in one pool of *Cx. pipiens* from Hohhot, Inner Mongolia. CxFVs were found in two pools of *Cx. tritaeniorhynchus* and 14 pools of *Cx. pipiens*, distributed in Hohhot, Inner Mongolia (11), Zaoyang, Hubei Province (1), Jining, Shandong Province (3), and Jin’an, Shanghai (1). JEV was found in three pools of *Cx. tritaeniorhynchus* from Zaoyang, Hubei (1) and Jinshan, Shanghai (2). HANKV was found in two pools of *Cx. pipiens* from Hohhot, Inner Mongolia. QBV was found in five pools of *Cx. pipiens*, two pools of *An. sinensis*, and 15 pools of *Cx. tritaeniorhynchus* from Jining, Shandong Province (4), Zaoyang, Hubei Province (4), Hohhot, Inner Mongolia (2), Sanya, Hainan Province (5), and Jinshan, Shanghai Municipality (7). YNCxFV was found in three pools of *Cx. tritaeniorhynchus* from Qionghai, Hainan Province. GETV was found in three pools of *Cx. pipiens*, one pool of *An. sinensis*, and one pool of *Cx. tritaeniorhynchus* from Hohhot, Inner Mongolia (3), Zaoyang County, Hubei Province (1), and Qionghai City, Hainan Province (1). The arbovirus distributions at each sentinel site are shown in **Figure 1**.

**Figure 2:**
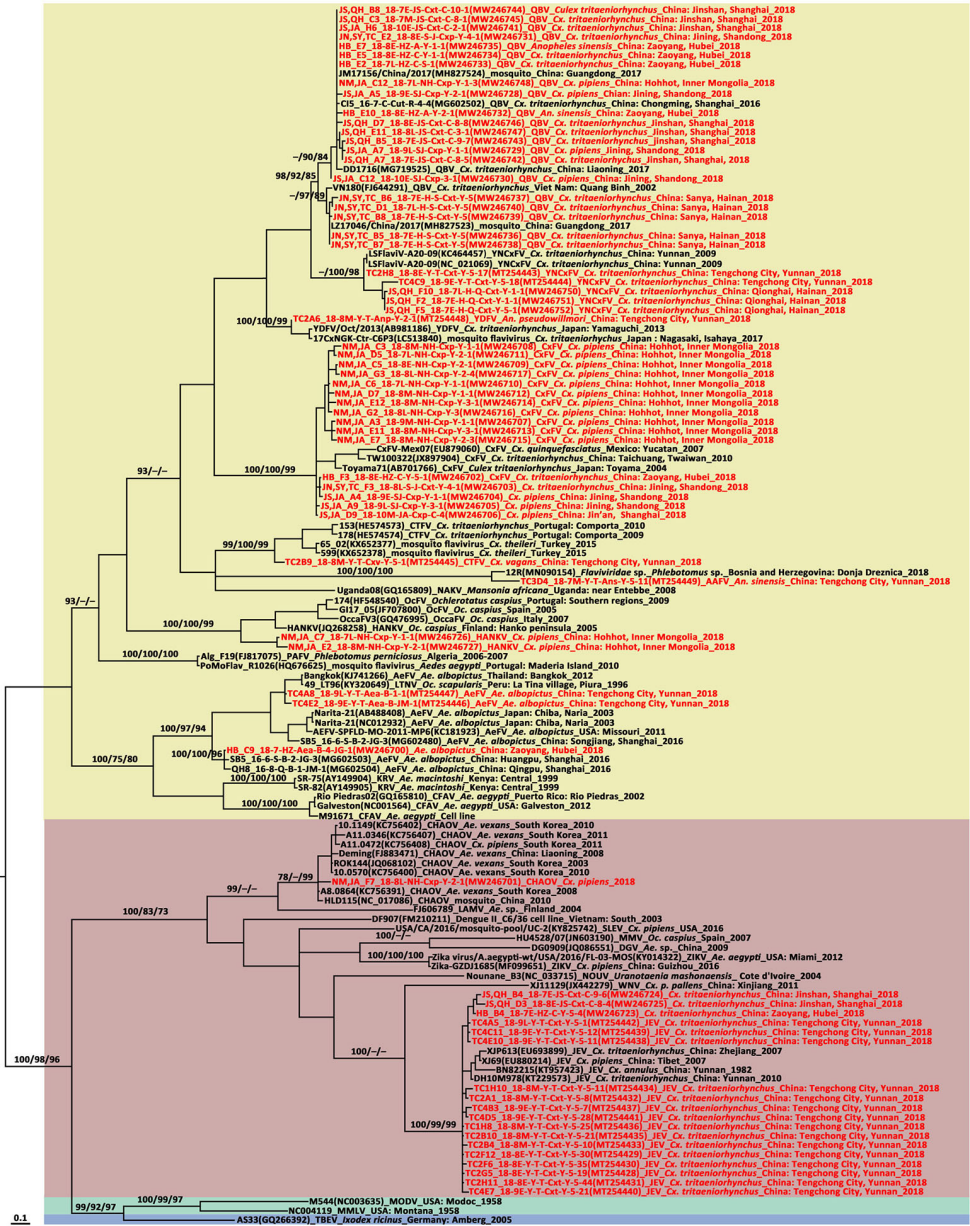
Phylogenetic tree generated by Bayesian analysis of partial non-structural five gene sequences of flavivirus. The virus strain, GenBank accession number, host, collection country and year are noted. The flavivirus sequences obtained in this study are marked in red. Bootstrap values (1,000 replicates, not shown for less than 75%) of maximum likelihood; Bayesian analyses and neighbor-joining are shown above the main lineages. The bar indicates 0.1 substitutions per site. Sequences shaded sky blue represent tick-borne flavivirus, those shaded aquamarine represent no-known vector flavivirus, those shaded khaki represent infect specific flavivirus, those shaded rose-brown represent mosquito-borne flavivirus. AAFV, Anopheles associated flavivirus; AeFV, Aedes flavivirus; CFAV, cell fusing agent virus; CTFV, Culex theileri flavivirus; CxFV, Culex flavivirus; DGV, Donggang virus; HANKV, Hanko virus; JEV, Japanese encephalitis virus; KRV, Kamiti River virus; LTNV, La Tina virus; MMLV, Montana myotis leukoencephalitis virus; MODV, Modoc virus; NAKV, Nakiwogo virus; PAFV, Phlebotomus associated flavivirus; QBV, Quang Binh flavivirus; OcFV, Ochlerotatus caspius flavivirus; SLEV, Santa Louis encephalitis virus; TBEV, tick-borne encephalitis virus; WNV, West Nile virus; YDFV, Yamadai flavivirus; YNCxFV, Yunnan Culex flavivirus.

The phylogenetic tree based on the *NS5* gene (**Figure 2**) showed that the *Flaviviridae* consisted of four clusters including mosquito-borne and tick-borne flaviviruses, no-known vector flaviviruses, and Insect specific flaviviruses (ISFVs). Each flavivirus had an independent lineage with high bootstrap values. Three mosquito-borne flavivirus-positive sequences were clustered in the JEV lineage while the other 44 sequences were scattered in the ISFV cluster. The CHAOV was genetically close to the mosquito-borne flavivirus clade and distant from the ISFV clade.

### Molecular Characterization and Phylogenetic Analysis Based on JEV *E* Genes

**Figure 3:**
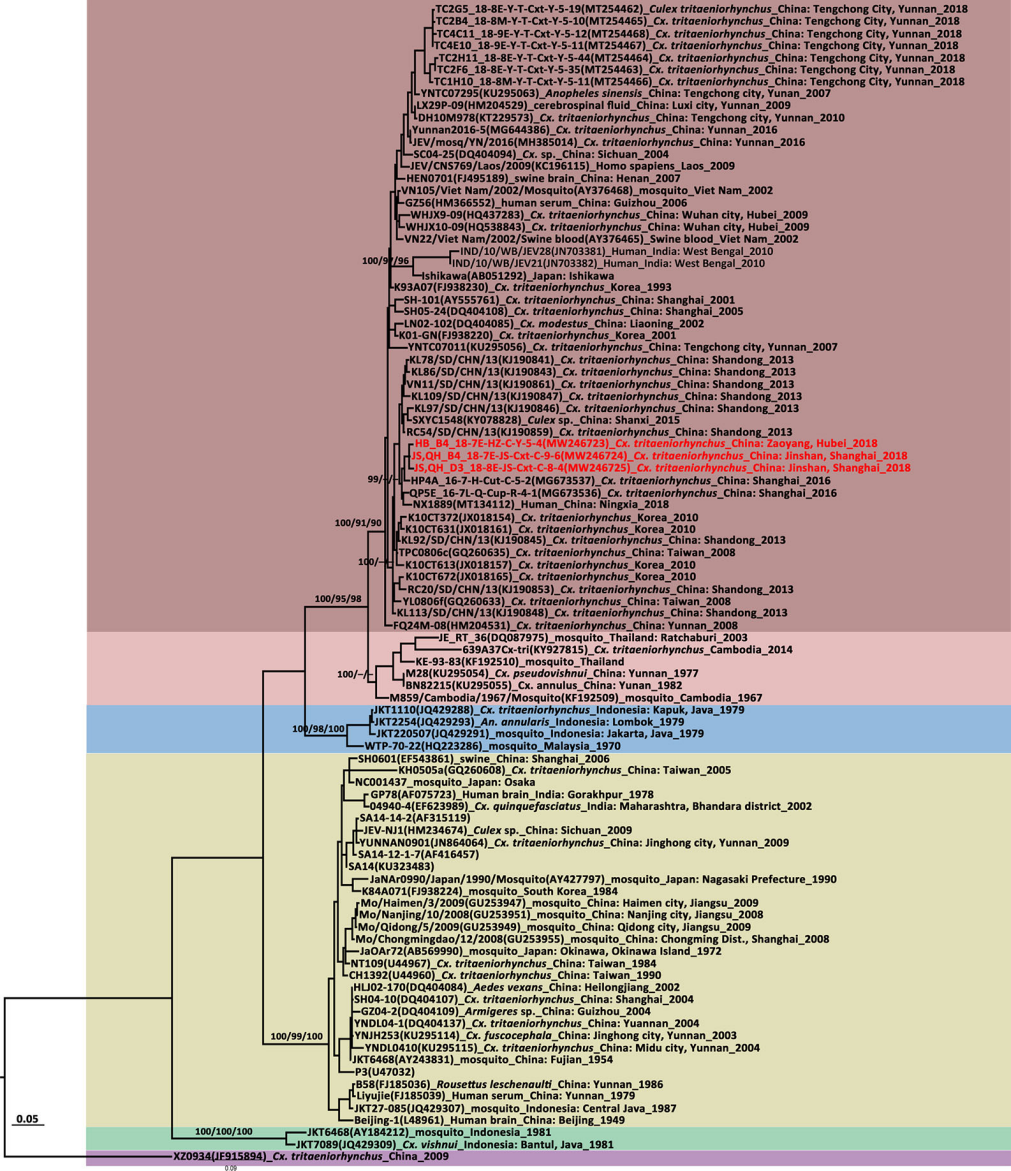
Maximum likelihood phylogenetic analysis of Japanese encephalitis virus (JEV) envelope gene sequences. The virus strain, GenBank accession number, host, collection country and year are noted. The JEV sequences obtained in this study are marked in red. The numbers above each branch represent the bootstrap support of the maximum likelihood, neighbor-joining, and Bayesian analyses, respectively, based on 1,000 replicates. The scale-bar indicates 0.05 substitutions per site. Sequences shaded misty rose represent the GI-a genotype, those shaded rose-brown represent the GI-b genotype, those shaded sky blue represent the GII genotype, those shaded khaki represent the GIII genotype, those shaded aquamarine represent the GIV genotype, and those shaded thistle represent the GV genotype.

For the JEV *E* gene, 99.36%–99.74% nucleotide sequence identity and 99.61%–100% amino acid sequence identity were determined for the newly detected Shanghai and Hubei strains. They had 87.76%–88.14% and 97.68%–97.76% similarity to the vaccine strain SA14-14-2 in terms of their nucleotide and amino acid levels, respectively. In the *E* gene tree (**Figure 3**), the newly detected Shanghai and Hubei strains fell into the GI-b cluster. They were genetically close and formed a cluster related to the strain obtained in Shanghai in 2016 (HP4A_16-7-H-Cut-C-5-2 strain, MT134112). However, they were distantly related to the strains circulating in Shanghai and Hubei before 2010. The deduced differences in amino acids among the E protein sequences were aligned to compare the newly detected strains against the vaccine strain (SA14-14-2) currently used in China. Relative to the SA14-14-2-derived strain (SA14), four amino acid substitutions were observed in the newly detected JEV strains, namely, E129 (Thr→Met), E222 (Ala→Ser), E327 (Ser→Thr), and E366 (Ala-Ser). There were no differences between the novel and vaccine strains in terms of their key amino acid sites related to antigenicity. All JEV strains carried the dominant haplotype SKSS based on the E123, E209, E227, and E408 sites in the E protein previously defined based on predicted positive selections (Han et al., 2014).

JEV *E* gene tree topologies (**Figure 3**) identified five major clades corresponding to genotypes I–V. GI comprised two distinct clades representing the GI-a and GI-b subgenotypes. All newly detected strains were clustered together and belonged to the GI-b genotype. They had high similarity and clustered with the sequences obtained in Shanghai in 2016 and the NX1889 strain (MT134112) isolated from a JE patient during an outbreak in Ningxia in 2018 (Liu et al., 2020b). A sequence comparison on JEV E gene revealed 98.51%–98.78% nucleotide and 99.61%–100% amino acid sequence identities between the newly obtained JEV strains and the NX1889 strain.

### Molecular Characterization and Phylogenetic Analysis Based on the GETV *E* Genes

**Figure 4:**
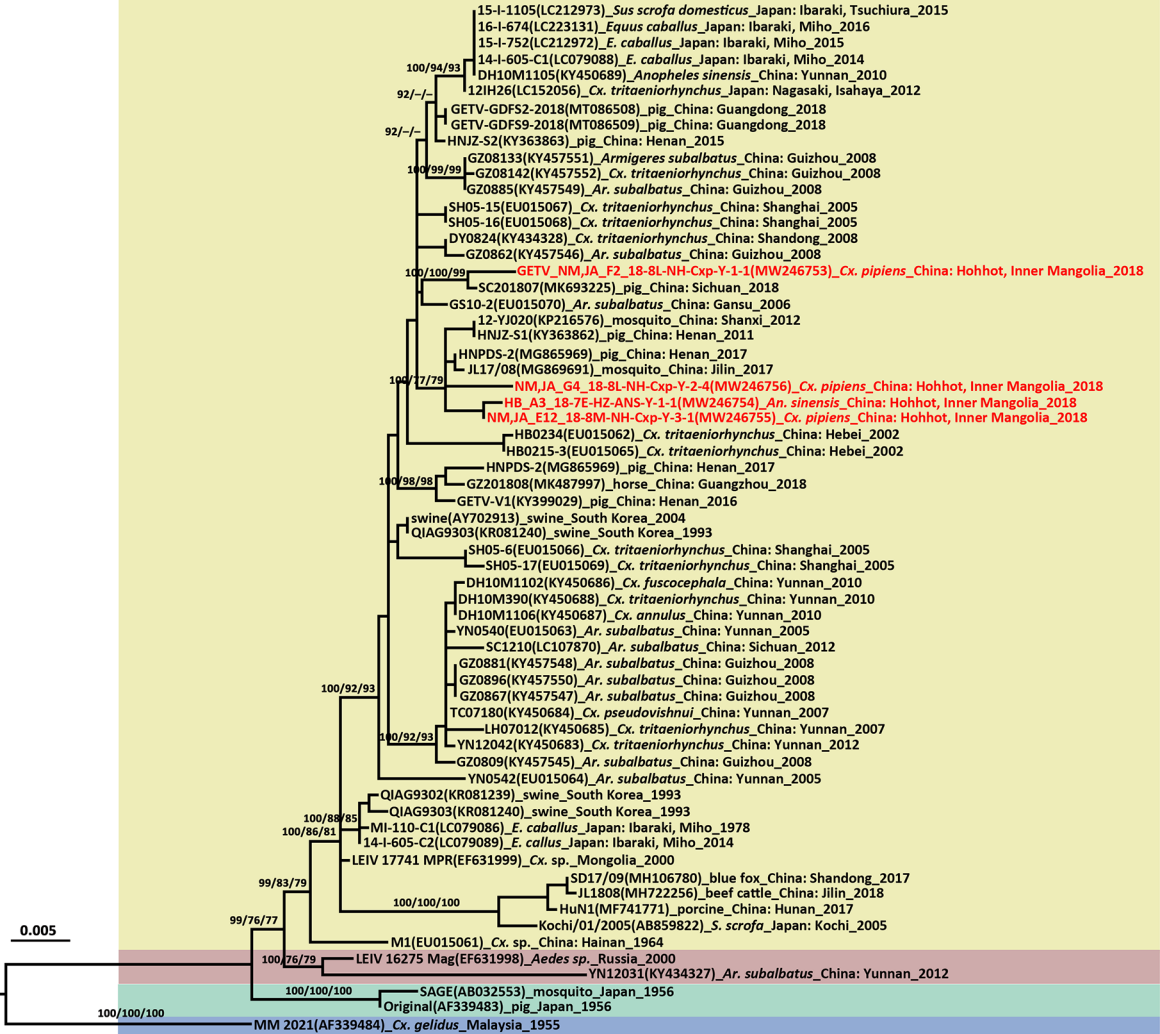
Phylogenetic tree generated by Bayesian analysis of Getah virus (GETV) envelope gene. The virus strain, GenBank accession number, host, collection country and year are noted. The GETV sequences obtained in this study are marked in red. Bootstrap values (1,000 replicates, not shown for less than 75%) of Bayesian analyses, maximum likelihood and neighbor-joining are shown above the main lineages. The scale-bar indicates 0.005 substitutions per site. Sequences shaded sky blue represent the GETV Group I, those shaded aquamarine represent Group II, those shaded khaki represent Group III, those shaded rose-brown represent Group IV.

The GETV *E2* gene was successfully amplified in four of five GETV positive pools. There were 98.50%–99.84% nucleotide and 98.82%–99.76% amino acid sequence identities among the newly detected Inner Mongolia and Hubei strains. The GETV strains identified here had 98.58%–98.74% nucleotide and 99.05%–99.53% amino acid sequence similarities to the vaccine strain MI-110 (Japan, LC079086) based on an analysis of the E2 protein-coding region sequences. The GETV *E2* gene tree topologies (**Figure 4**) identified four distinct clades corresponding to groups I–IV. The newly obtained strains fell into two sublineages of Group III. The HB_A3_18-7E-HZ-ANS-Y-1-1, NM,JA_E12_18-8M-NH-Cxp-Y-3-1, and NM,JA_G4_18-8L-NH-Cxp-Y-2-4 strains were close to those identified in swine (HNPDS-2) and mosquito (JL17/08) samples collected in Henan and Jilin Provinces. The NM,JA_F2_18-8L-NH-Cxp-Y-1-1 strain was close to the swine sample (SC201807) obtained in Sichuan. The three GETV strains from Inner Mongolia were distributed in two sublineages and were distantly related to the strain from neighboring Mongolia. GETV was first detected in a single pool of *Cx. pipiens* (NMDK1813-1 strain) collected in Inner Mongolia in 2018 (Cheng et al., 2020). The NMDK1813-1 strain was close to the GS10-2 (EU015070) strain from Gansu Province (Cheng et al., 2020). GETV was detected in samples from Hubei (ES26) (Gao et al., 2015) collected in 2010. The phylogenetic analysis suggests that the ES26 strain was genetically close to HB0234 strain from Hubei (Gao et al., 2015). It’s a pity that there were no available NMDK1813-1 strain and ES26 strain sequences available on GenBank.

The GETV *E2* gene is 1,266 nt long and encodes a 422-aa glycoprotein. The deduced amino acid differences between the E2 protein sequences and those of the vaccine derived strain (MI-110) currently used in Japan were aligned and compared. Six amino acid residues in the newly detected GETV strains differed from those of the MI-110 strain, namely, E75 (Met→Ile), E86 (His→Tyr), E97 (Met→Ile), E194 (Glu→Gly), E248 (Leu→Ser), and E323 (Asp→Glu). Comparison of the E2 protein amino acids showed that Gly at locus 194 was unique to MI-110 whereas the Glu substitution at E194 (Glu→Gly) was observed in all other strains.

The length of the entire genome of the newly detected GETV (NM,JA_F2_18-8L-NH-Cxp-Y-1-1 strain, MT254427) was 11,689 bp. It comprised 5’UTR (78 nt), two ORFs encoding non-structural proteins (7,404 nt) and structural proteins (3,762 nt), 3’UTR (401 nt), and a 26S RNA junction region located between the non-structural and structural protein coding regions (44 nt; 7,483–7,526). The complete genome was amplified with 11 pairs of overlapping primers (**Table S2**). The virus isolated from *Cx. pipiens* had 99.73% and 99.84% similarities at the nucleotide and amino acid levels, respectively, to the SC201807 strain isolated from mixed infected pig reproductive and respiratory syndrome blood in Sichuan in 2018 (Jiang et al., 2019).

### Molecular Characterization and Phylogenetic Analysis Based on the CHAOV *NS5* Genes

In the flavivirus *NS5* tree, CHAOVs distributed in the mosquito-borne flavivirus clade and were remote from ISFVs. CHAOVs were previously detected in Liaoning Province, China (Wang et al., 2009). This record of CHAOV is the first for Inner Mongolia.

The complete genome of the NM,JA_F2_18-8L-NH-Cxp-Y-1-1 strain was 10,679 nt and consisted of an ORF encoding 3,436 amino acids flanked by 99 and 272 nucleotides at the 5′UTR and 3′UTR ends, respectively. The complete genome was amplified with 11 pairs of overlapping primers (**Table S3**).

Compared to the genome sequences of other CHAOV strains, the newly discovered NM,JA_F2_18-8L-NH-Cxp-Y-1-1 strain had 98.91% identity to HLD115 (NC_017086) detected in mosquitoes from Hulu Island, Liaoning Province, China in 2010. It had 97.90% nucleotide similarity to the prototype Deming (FJ883471) strain from Liaoning, China and 97.87% nucleotide similarity to the ROK144 strain (JQ068102) from South Korea. Similarity of the four CHAOV strains to the whole genome available on GenBank was 99.7%–99.9% at the amino acid level.

### Sequence Analysis and Phylogenetic Characterization of ISFVs

**Figure 5:**
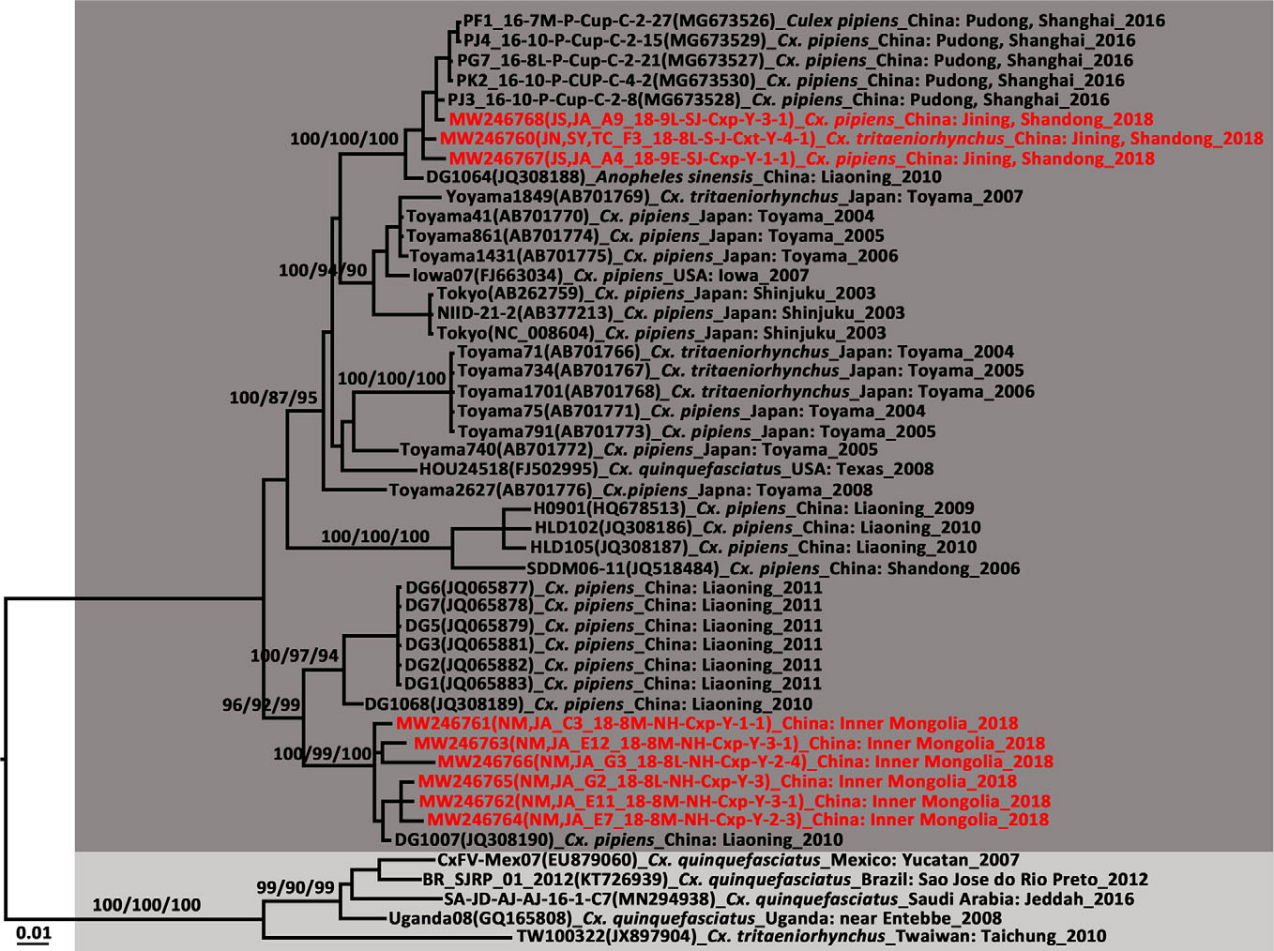
Phylogenetic tree generated by Bayesian analysis of Culex flavivirus (CxFV) complete genome. The virus strain, GenBank accession number, host, collection country and year are noted. The CxFV sequences obtained in this study are marked in red. Bootstrap values (1,000 replicates, not shown for less than 75%) of Bayesian analyses, maximum likelihood and neighbor-joining are shown above the main lineages. The scale-bar indicates 0.01 substitutions per site. Dark grey and light grey indicate CxFV Asian/USA genotype and Africa/Caribbean/Latin America genotype, respectively.

AeFV was detected in a pool of *Ae. albopictus* from Zaoyang, Hubei Province. Phylogenetic analysis revealed a close genetic relationship between it and the strains from Shanghai. This AeFV record was the first for Hubei Province. In China, AeFVs were already detected in mosquito samples collected in Shanghai Municipality in 2016 (Fang et al., 2018) and Yunnan Province in 2018 (Fang et al., 2021b). Sixteen CxFVs were observed at four sentinel sites. The CxFV *E* gene was successfully amplified in nine strains. The CxFVs in the *E* gene phylogenetic tree (**Figure 5**) were divided into two genotypes. All newly detected CxFVs were clustered in the Asia/USA genotype clade whereas those obtained from Shandong and Shanghai were distantly related to those from Inner Mongolia. To the best of our knowledge, these records of CxFV are firsts for Hubei and Inner Mongolia. The whole JS,JA_A4_18-9E-SJ-Cxp-Y-1-1 genome was 10,799 bp long and comprised an ORF encoding 3,364 amino acids flanked by 71 and 636 nucleotides at the 5′UTR and 3′UTR ends, respectively. Eleven overlapping primers (**Table S4**) amplified the complete CxFV genome. A genome-wide comparison showed that the JS,JA_A4_18-9E-SJ-Cxp-Y-1-1 strain from Shandong Province had 99.6% nucleotide and 99.67% amino acid identities with the DG1064 strain (JQ308188.1) from *An. sinensis* in Liaoning Province.

Two *Cx. pipiens* pools from Inner Mongolia were positive for HANKV. They had 98.09% similarity with each other and shared 80.47%–87.88% nucleotide sequence identity with the partial *NS5* genes of strains from Europe. This HANKV record is a first for China. It was already detected in Finland, Spain, Italy, and Portugal (Blitvich and Firth, 2015). Hence, this virus has a broad geographical distribution and a wide host range.

QBVs have been detected at five sentinel sites in this study including Shandong, Hubei, Hainan, Inner Mongolia, and Shanghai. This record of QBV was a first for all the aforementioned sites except Shanghai. The *NS5* gene sequence similarity was 86.36%–100% among QBV strains. The strains JS,JA_A5_ 18-9E-SJ-Cxp-Y-2-1 from Jining and QBV_JN,SY,TC_B6_18-7E-H-S-Cxt-Y-5 from Sanya City had only 87.50% similarity. QBVs in the *NS5* tree were divided into two clusters. The strains from Sanya City in Hainan Province gathered with the QBV prototype strain from Vietnam. The strains from Hubei, Shanghai, Inner Mongolia, and Shandong clustered with that from Shanghai. JM17156 (MH827524) and LZ17046 (MH827523) were isolated from mosquitoes collected in Guangdong but had only 91.60% nucleotide sequence similarity and were remote from each other in the phylogenetic tree. The boundary of the two QBV lineages is probably located between 21°N and 22°N.

We elucidated the complete genome of the novel JS,JA_H6_18-10E-JS-Cxt-C-2-1 strain. It was 10,785 nt long and had an ORF encoding 3,360 amino acids flanked by 85 and 620 nucleotides at the 5′UTR and 3′UTR ends, respectively. The complete genome was amplified with 10 pairs of overlapping primers (**Table S5**). A whole-genome sequence comparison of JS,JA_H6_18-10E-JS-Cxt-C-2-1 and the other four representative QBV strains showed 88.4%–99.5% nucleotide sequence identity. The deduced amino acid sequence identities were in the range of 97.1–99.8%. JS,JA_H6_18-10E-JS-Cxt-C-2-1 had 99.5% genetic identity with the DD1716 strain detected in *Cx. tritaeniorhynchus* in Dandong City, Liaoning Province, China. However, it had only 89.79% nucleotide sequence identity with the VN180 strain.

Three newly identified YNCxFVs were obtained from three pools of *Cx. tritaeniorhynchus* collected in Qionghai, Hainan Province, China and they had 98.47%–98.85% nucleotide identity. This YNCxFV record is the first outside Yunnan Province. The phylogenetic analysis showed that the newly detected strains were close to those obtained from *Cx. tritaeniorhynchus* in Tengchong County, Yunnan Province in 2018. The partial *NS5* gene had 90.53%–100% nucleotide sequence identity with those for YNCxFV in GenBank.

### Arbovirus Infection Rates in Mosquitoes

The arbovirus infection rates in the mosquitoes collected from the sentinel sites were estimated by bias-corrected MLE and MIR and are shown in **Table 2**. For JEV, the overall bias-corrected MLE values expressed as number of infected mosquitoes per 1,000 *Cx. tritaeniorhynchus* were 4.52 (0.80–14.64) and 0.35 (0.02–1.69) in Jinshan and Zaoyang, respectively. The GETV MLE per 1,000 individuals were 0.14 (0.04–0.37, *Cx. pipiens*), 3.72 (0.21–17.36, *An. sinensis*), and 0.75 (0.04–3.60, *Cx. tritaeniorhynchus*) in Hohhot, Zaoyang, and Qionghai, respectively. JEV and GETV were detected in samples collected in July and August 2018.

**Table 2:** Bias-corrected maximal likelihood estimations (MLE) and minimum infection rates (MIR) of mosquito-borne flaviviruses at various sentinel sites during the mosquito activity seasons of 2018.

Survey areas	Detected virus	host	No. individuals	No. PP	No. pools	Positive pool rate (%)	Bias Corrected MLE (95% *CI*)	MIR (95% *CI*)
Hohhot, Inner Mongolia	GETV	*Culex pipiens*	22049	3	225	1.33	0.14 (0.04–0.37)	0.14 (0.00–0.29)
	CHAOV	*Cx. pipiens*	22049	1	225	0.44	0.05 (0.00–0.22)	0.05 (0.00–0.13)
	CxFV	*Cx. pipiens*	22049	11	225	4.89	0.51 (0.27–0.88)	0.50 (0.20–0.79)
	QBV	*Cx. pipiens*	22049	2	225	0.89	0.09 (0.02–0.30)	0.09 (0.00–0.22)
	OcFV	*Cx. pipiens*	22049	2	225	0.89	0.09 (0.02–0.30)	0.09 (0.00–0.22)
Zaoyang, Hubei	GETV	*Anopheles sinensis*	273	1	28	3.57	3.60 (0.21–17.36)	3.66 (0.00–10.83)
	JEV	*Cx. tritaeniorhynchus*	2475	1	83	1.20	0.35 (0.02–1.69)	0.35 (0.00–1.83)
	QBV	*Cx. tritaeniorhynchus*	2475	2	83	2.41	0.70 (0.13–2.28)	0.70 (0.00–1.66)
	QBV	*An. sinensis*	273	2	28	7.14	7.12 (1.34–22.69)	7.33 (0.00–17.44)
	CxFV	*Cx. tritaeniorhynchus*	2475	1	83	1.20	0.35 (0.02–1.69)	0.35 (0.00–1.03)
	AeFV	*Aedes albopictus*	152	1	40	2.50	6.50 (0.38–30.94)	6.58 (0.00–19.43)
Jining, Shandong	QBV	*Cx. tritaeniorhynchus*	217	1	16	6.25	4.35 (0.27–20.66)	4.61 (0.00–13.62)
	QBV	*Cx. pipiens*	146	3	20	15.00	21.53 (5.89–57.53)	20.55 (0.00–43.56)
	CxFV	*Cx. tritaeniorhynchus*	217	1	16	6.25	4.38 (0.27–20.89)	4.61 (0.00–13.62)
	CxFV	*Cx. pipiens*	146	2	20	10.00	14.13 (2.59–46.19)	13.70 (0.00–32.55)
Jinshan, Shanghai	JEV	*Cx. tritaeniorhynchus*	452	2	69	2.90	4.48 (0.80–14.64)	4.42 (0.00–10.54)
	QBV	*Cx. tritaeniorhynchus*	452	2	69	2.90	16.27 (7.24–31.94)	15.49 (4.10–26.87)
Jing’an, Shanghai	CxFV	*Cx. pipiens*	208	1	69	1.45	4.93 (0.28–24.22)	4.81 (0.00–14.21)
Qionghai, Hainan	GETV	*Cx. tritaeniorhynchus*	1351	1	44	2.27	0.75 (0.04–3.60)	0.74 (0.00–2.18)
	YNCxFV	*Cx. tritaeniorhynchus*	1351	3	44	6.82	2.22 (0.60–5.95)	2.21 (0.00–4.71)
Sanya, Hainan	CxFV	*Cx. tritaeniorhynchus*	191	5	10	50.00	61.25 (21.50–248.10)	26.18 (3.53–48.82)

AeFV, Aedes flavivirus, CHAOV, Chaoyang virus; CI, confidence interval; CxFV, Culex flavivirus; GETV, Getah virus; HANKV, Hanko virus; JEV, Japanese encephalitis virus; gene, PP, positive pool, YNCxFV, Yunnan Culex flavivirus.

Bias-corrected MLE for the CHAOV, AeFV, and YNCxFV infection rates were 0.05 (0.00–0.22), 6.50 (0.38–30.94), and 2.22 (0.60–5.95) per 1,000 individuals in Hohhot, Zaoyang, and Qionghai, respectively. Bias-corrected MLE for the CxFV and QBV infection rates were in the ranges of 0.35–14.13 and 0.09–61.25, respectively.

## Discussion

Climate change, biogeography, and human and avian behavior have been implicated in the spread of arbovirus. To the best of knowledge, our study is the first to record HANKV in China. Moreover, this work research reports for the first time the presence of AeFV, CHAOV, CxFV, QBV, and YNCxFV at multiple sentinel sites.

Hohhot, Inner Mongolia had a high mosquito density and the richest arbovirus diversity in this study. GETV, CxFV, HANKV, QBV, and CHAOV were detected at this location. Zaoyang was second to Hohhot in terms of mosquito abundance and it harbored JEV, GETV, AeFV, QBV, and CxFV. Tengchong in Yunnan Province was another sentinel site of the mosquito-borne disease surveillance in 2018. For a putative focal point of a JE epidemic has been found there, the results were published separately (Fang et al., 2021b).

### JEV

As JEV naturally circulates, JE outbreaks sporadically occurred in China. The main reason for the observed increase in the number of reported JE cases between 2016 and 2018 was that the incidence rose in the subpopulation aged ≥ 40 years (Wu et al., 2020). Furthermore, the JE outbreaks occurred mainly in the north-central regions but not in the provinces wherein JEV was strongly endemic as of the early 2000s (Zheng et al., 2012).

A phylogenetic analysis of the *E* gene showed that JEV is divided into the GI–GV genotypes. GI is further subdivided into GI-a and GI-b. The latter gradually replaced GIII over the past 30 years (Schuh et al., 2014; Do et al., 2016). GI was more efficiently amplified than GIII in mosquito and porcine cells whereas the latter was more efficiently amplified in human rhabdomyosarcoma clones (Han et al., 2014). However, GI has been implicated in three recent human JE outbreaks. A few cases in the JE outbreak in India in 2010 were associated with GI but most involved GIII (Sarkar et al., 2012; Han et al., 2014). The JEV strain isolated from *Cx.* spp. during the 2010 JE outbreak in South Korea belonged to GI, although no human sequences were available (Seo et al., 2013). JEVs isolated from human and *Cx. tritaeniorhynchus* samples collected during the 2018 outbreak in Ningxia, China belonged to GI-b but no GIII was detected (Liu et al., 2020b). The phylogenetic tree based on the *E* gene demonstrated that the newly detected JEV strains gathered with the Ningxia strain and there were no amino acid substitutions on the E protein. High nucleotide identities were observed among the JEV strains obtained from Ningxia, Hubei, and Shanghai. Hence, frequent, long-range JEV transmission has occurred within China probably *via* migratory birds and windblown mosquitoes. The E proteins in the newly detected JEV strains differed from that in the live attenuated SA14-14-2 vaccine at E129 (Thr→Met), E222 (Ala→Ser), E327 (Ser→Thr), and E366 (Ala-Ser). No divergences were detected among them in terms of their key amino acid sites related to antigenicity. The E protein is a major constituent of the mature virion surface and is under constant selection pressure. It plays critical roles in infectivity and immunity (Schuh et al., 2014). It remains to be determined whether these mutations are associated with host adaptation. The vaccines currently used in China are derived from GIII. They confer protection against GI-GIV but not against GV (Cao et al., 2016). Thus, surveillance must be sustained to characterize circulating JEVs on a genetic level and avoid potential vaccine failure.

The JEV infection rate in *Cx. tritaeniorhynchus* from Jinshan District, Shanghai Municipality was 4.52/1,000 (0.80–14.64). The prevalence of West Nile virus (WNV) constituting an “epidemic risk” was > 5/1,000 mosquitoes (Tao et al., 2014). There were no data for JEV. Thus, considering the data of WNV, the Jinshan District is at potential risk of JEV epidemics.

As JEV constantly circulates in nature, it continues to pose a serious threat to public health. The vaccination program has altered the JEV endemic status and populations at risk in various areas. Hence, the JEV prevention strategy is suggested to be modified accordingly. Consistent immunization programs are required for children. Targeted JE vaccination should be administered to adults in areas to which JE is highly endemic. Domestic pigs must be relocated to communal facilities away from human habitation and irrigated rice production regions. The living conditions of people in remote rural areas require improvement to reduce the risk of viral spillover from the animal reservoir to the human population.

### GETV

GETV was first isolated from *Cx. gelidus* in Malaysia in 1955 (Karabatsos, 1985). Phylogenetic analyses suggested that GETV emerged ~145 years ago and gradually evolved into four groups (Li et al., 2017b). GETV is active in Southeast Asia and Eurasia and transmitted by *Cx.* spp. to pigs and horses (Li et al., 2017b). There were frequent GETV outbreaks in Japan during the 1980s (Kamada et al., 1980). An inactivated whole-virus vaccine derived from MI-110 (Nisseiken, Japan) isolated in 1978 was developed. Two vaccine doses were administered to racehorses in Japan to prevent GETV infection (Bannai et al., 2015). However, unexpected GETV outbreaks occurred in vaccinated racehorses in Miho in 2014–2015 (Nemoto et al., 2015; Bannai et al., 2016). The reason was mainly for a single vaccine dose might not suffice to protect racehorses in areas with high GETV infection rates (Bannai et al., 2015; Bannai et al., 2017). There have been seven major GETV outbreaks. Five involved racehorses in Japan (Kamada et al., 1980; Bannai et al., 2015; Bannai et al., 2016), one affected horses in India (Brown and Timoney, 1998), and one concerned pigs in China in 2017 (Yang et al., 2018).

**Figure 6:**
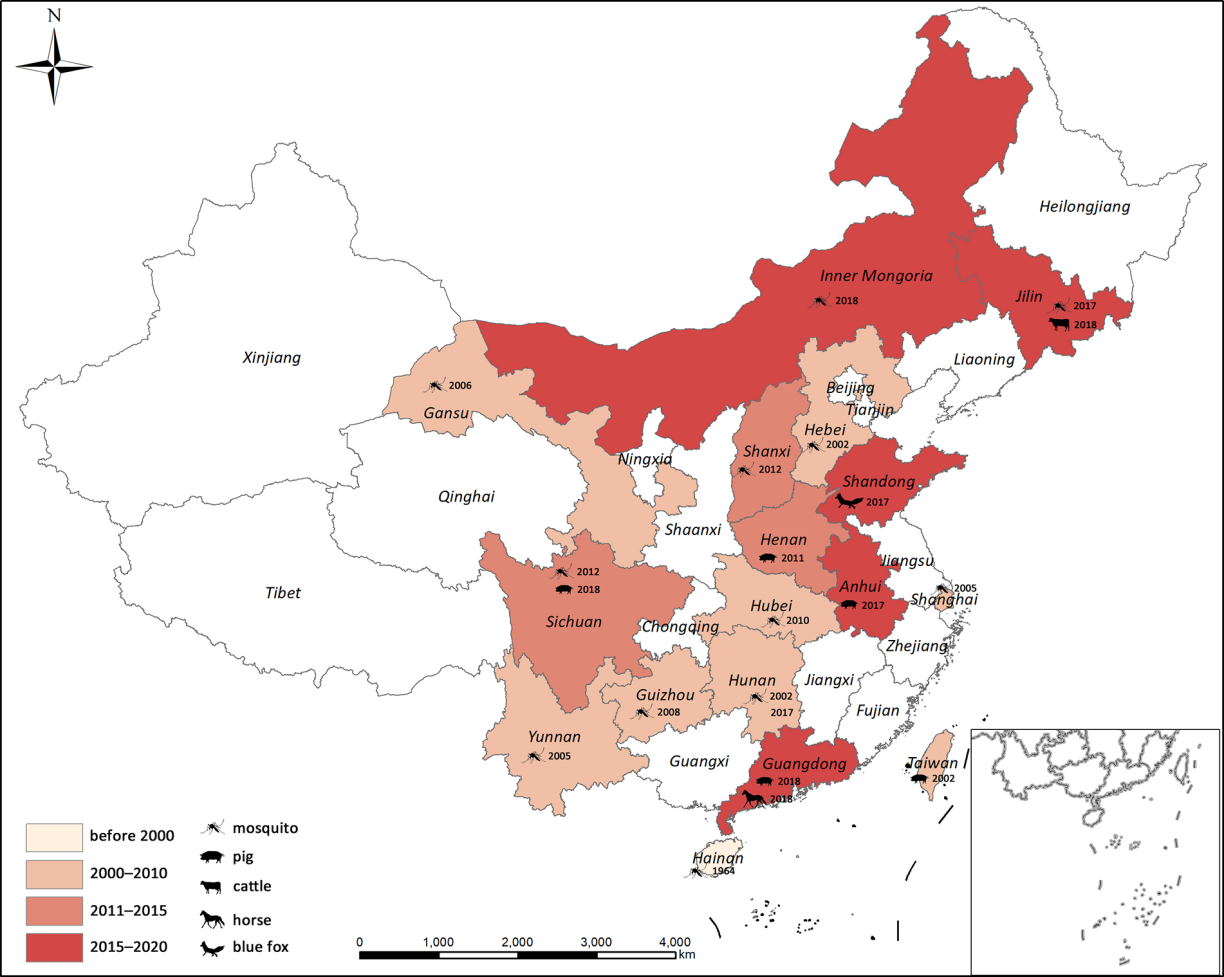
Geographical distribution of GETV in China. Years correspond to the first GETV detection in each province.

In China, GETV was first isolated from *Cx*. sp. in Hainan Province, in 1964 (Li et al., 1992). It was detected in mosquitoes from Hebei (2002), Shanghai (2005), Yunnan (2005), Gansu (2006, (Zhai et al., 2008)), Guizhou [2008, (Li et al., 2017b)], Hubei [2010, (Gao et al., 2015)], Shanxi [2012, (Zheng et al., 2015)], Sichuan [2012, (Li et al., 2017a)], Jilin [2017, (Liu et al., 2019)], and Inner Mongolia [2018, (Cheng et al., 2020)]. The first vertebrate GETV was isolated in Taiwan in 2002 (Chang et al., 2006). The number of cases of GETV-affected mammals has rapidly increased over the last decade. These included pigs in Henan [2011, (Zhou et al., 2018)], Hunan [2017, (Yang et al., 2018)], Anhui [2017 (available on GenBank but unpublished)], Sichuan [2018, (Jiang et al., 2019)], and Guangdong [2018, (Xing et al., 2020)], blue foxes in Shandong [2017, (Shi et al., 2019)], beef cattle in Jilin [2018, (Liu et al., 2019)], and horses in Guangdong [2018, (Lu et al., 2019)]. A GETV outbreak occurred on a swine farm in Hunan Province in June and July 2017 and caused many fetal mummies, stillbirths, and piglet deaths (Yang et al., 2018). In contrast, mosquito-borne GETV has not yet been recorded in Hunan. Sudden onset of fever, caused by GETV, was reported for racehorses at a training center in Guangdong in 2018, while no GETV infection was detected in archived equine serum samples collected between 2014 and 2018 (Lu et al., 2019). To date, GETV records have covered 17 provinces in China (**Figure 6**) and most of them were reported within the last decade. Several serological surveys revealed widespread GETV occurrence in humans and domestic animals in the Asia-Pacific region (Fukunaga et al., 2000; Liang et al., 2010; Li et al., 2018). Frequent GETV detection in various vertebrates and mosquitoes over a wide geographical area suggests that this virus may cause an epidemic in China. Thus, it is important to conduct research on the molecular evolution and infection rate dynamics of GETV in its vectors and natural hosts.

The GETV *E2* phylogenetic tree revealed that vector and amplifying host sequences were in close proximity to each other. Thus, there is a potential risk of GETV epidemics in domestic animals. In addition, the GETV strains collected within the same province were distributed in two distinct Group III clusters. For this reason, GETV is frequently transmitted in China. The GETV infection rates in *Cx. tritaeniorhynchus* were 2.31/1,000 and 1.60/1,000 when the pathogen was detected in blue fox (Shi et al., 2019) and beef cattle (Liu et al., 2019), respectively. In Zaoyang, the GETV infection rate was 3.60/1,000 *An. Sinensis.* Inner Mongolia has the largest grassland in China and animal husbandry is a major industry there. This region raises domestic livestock such as cattle, sheep, swine, and horses. It is at high risk of a GETV epidemic as it has a high concentration of GETV vectors and host animals. All three GETV strains from Inner Mongolia were observed in *Cx. pipiens*. This mosquito species breeds in dirty water and is common in urban and suburban areas. Hence, *Cx. pipiens* harboring GETV intensify its potential threat to the public health.

GETV is prevalent and widespread in China. However, its harm to livestock may have been minimized as most mammalian GETV cases are subclinical. Serious clinical GETV symptoms and death have occurred in piglets (Yang et al., 2018) and ponies (Bannai et al., 2015; Bannai et al., 2016). The presence of GETV on pig breeding farms adjacent to horse and cattle farms merits close attention as pigs may amplify GETV in nature (Kumanomido et al., 1988). In addition, it is urgently required to develop a universally applicable vaccine for the prevention and control of GETV in domestic animals in China.

### ISFVs

The ISFVs detected in the present study included AeFV, CHAOV, CxFV, HANKV, QBV, and YNCxFV and most of them were new local records. The CHAOV sister to Lammi virus (LAMV) clustered with the mosquito-borne human flavivirus pathogens and were distant from the ISFV clade (**Figure 2**). CHAOV was first isolated from *Ae. vexans* in Liaoning, China in 2008 (Wang et al., 2009). It was then detected in *Ae. vexans*, *Ae. albopictus*, *Cx. pipiens*, and *Ar. subalbatus* in South Korea during vector surveillance between 2008 and 2011 (Takhampunya et al., 2014). Here, it was also found in Inner Mongolia. The three CHAOV records ranged from 35°N to 42°N. Extremely low sequence diversity between the CHAOV strains and the whole genome available in GenBank suggests that CHAOV only recently spread to Northeast Asia, has a brief history, and is evolving slowly. CHAOV may only replicate in mosquito vectors and not in mammalian or avian hosts (Lee et al., 2013). Similar pathological traits were reported for LAMV (Huhtamo et al., 2009), Nounane (NOUV) (Junglen et al., 2009), and Marisma mosquito (MMV) (Vazquez et al., 2012) viruses infecting various cell lines. It is unknown why CHAOV, LAMV, Donggang virus, NOUV, and MMV phylogenetically resemble mosquito-borne zoonotic flaviviruses but have no recognized vertebrate hosts.

Phylogenetic analysis of the CxFV *E* gene demonstrated that the newly detected CxFVs belong to the Asia/USA genotype but clustered in two lineages (**Figure 5**). The Shanghai and Jining strains were close to the Shanghai strain detected in 2016. The Inner Mongolia strains were clustered and phylogenetically distant from the others. The CxFV *E* tree (**Figure 5**) revealed that the Asia/USA genotype was localized mainly above 30°N latitude. The zone between 30°N and 32°N is the boundary of *Cx. pipiens* and *Cx. quinquefasciatus* in China (Lu, 1997). The Africa/Caribbean/Latin American genotype was found in *Cx. quinquefasciatus* from Mexico, Brazil, and Saudi Arabia. CxFV has not yet been isolated from *Cx. quinquefasciatus* in mainland China. Here, CxFV was not found in *Cx. quinquefasciatus* collected from Hainan or Yunnan Province. QBV was first isolated from samples collected in Vietnam in 2002 (Crabtree et al., 2009). It was then detected in mosquito samples collected from Chongming Island in Shanghai Municipality in 2016 (Fang et al., 2018). Here, QBV was observed at Jining, Zaoyang, Sanya City, Jinshan, and Hohhot. Hence, it is widespread in China. The primary QBV vector is *Cx. tritaeniorhynchus*. In this study, it was also detected in *An. sinensis* and *Cx. pipiens*. In previous studies (Zuo et al., 2014; Fang et al., 2021b), YNCxFV was restricted to Yunnan Province. In the present study, it was also detected in *Cx. tritaeniorhynchus* from Qionghai, Hainan Province. The taxonomic value of YNCxFV is debatable as the nucleotide identity between YNCxFV and QBV was only 83% (Zuo et al., 2014). The threshold for members species of *Flavivirus* was 84% (Kuno et al., 1998). For the partial *NS5* gene, the lowest intraspecies QBV identity was 87.50%. The interspecies similarity between QBVs and YNCxFVs was in the range of 82.40–87.88%. In the *NS5* phylogenetic tree (**Figure 2**), the YNCxFV sequences formed a sister lineage to the group containing the QBV sequences. Moreover, both species overlapped in terms of geographic distribution and mosquito host. Thus, it remains to be established *via* cytopathy and neutralization tests whether YNCxFV is an independent ISFV species. AeFV has been widespread in Japan, Thailand, USA, Peru, and Italy (Blitvich and Firth, 2015). In China, it was first detected in samples collected from Shanghai in 2016 (Fang et al., 2018). It was then observed in Yunnan (Fang et al., 2021b) and Hubei in 2018. *Aedes albopictus* is the primary vector of AeFV. HANKVs were distributed in Finland, Spain, Italy, and Portugal (Blitvich and Firth, 2015). The present study was the first to report HANKVs in *Cx. pipiens* in Asia. There is broad genetic diversity among HANKV strains (83.52%–98.09%) as they have a wide geographic span. The wide dispersion and host range of ISFVs support the hypothesis that ISFV transmission in sympatric species may be linked to a common infection source such as food (Calzolari et al., 2016).

The main limitation of our study was that no mosquito virus homogenate supernatants were cultivated in mosquito or vertebrate cell lines. The low viral titers measured in the mosquito homogenates may have skewed the pathogen detection data. Consequently, the real natural arbovirus infection rates may have been underestimated. In future work, we will try to isolate them and conduct further analysis.

In conclusion, monitoring the presence of viral pathogens in mosquitoes can forecast the risks of arbovirus epidemics. The newly detected JEV strains and the Ningxia strain were 100% identical in terms of their E protein at the amino acid level. The JEV infection rate in Jinshan District approached epidemic proportions. GETV was prevalent in more than half the provinces of China. Mammalian GETV infection and outbreaks have been reported in recent years. Hence, attention must be paid to the putative threat that GETV could pose to animal and human health. Reports of ISFV cases have dramatically increased in recent decades. It is now known that ISFVs have wide geographic and host ranges. Though it appears that ISFVs can only replicate in mosquito cells, their possible roles in mammalian pathogenesis cannot be ruled out. It also remains to be determined whether ISFV-infected mosquitoes escape vector competence or pathogenic flavivirus superinfection can occur. The present study showed that wide-ranging, systematic, and continuous monitoring of mosquito-borne circulating viruses is urgently needed in China. This surveillance program could elucidate viral diversity, geographic distribution, evolution, genotype shift, and infection rates. It would also facilitate accurate and timely estimates of actual pathogen burdens and predict the prevalence of dengue and other emerging and existing mosquito-borne pathogens.

## Data Availability Statement

The original contributions presented in the study are included in the article/**Supplementary Material**. Further inquiries can be directed to the corresponding author.

## Author Contributions

YF: conceived the study, performed molecular work, data analysis, and write the manuscript. WZ: performed molecular work. J-BX: drew map of mosquito collection sites for the virus detection. YZ: conceived the study and performed data analysis. All authors contributed to the article and approved the submitted version.

## Funding

This research was funded by “The Special Foundation of Basic Science and Technology Resources Survey of Ministry of Science and Technology of China (No. 2017FY101203)”, “The Fifth Round of Three-Year Public Health Action Plan of Shanghai (No. GWV-10.1-XK13)”, and “The Project of Basic Platform of National Science and Technology Resources of the Ministry of Sciences and Technology of China (No. TDRC-2019-194-30)”.

## Conflict of Interest

The authors declare that the research was conducted in the absence of any commercial or financial relationships that could be construed as a potential conflict of interest.
