# Supplementary material for: Monitoring Mosquito-Borne Arbovirus in Various Insect Regions in China in 2018
Source: Front Cell Infect Microbiol. 2021 Mar 11;11:640993. doi: 10.3389/fcimb.2021.640993 (PMC8006455; doi:10.3389/fcimb.2021.640993)
Supplement: Supplementary file 1 [file Table_1.docx]

Supplementary Material

# Supplementary Tables

**Table S1. Summary of adult mosquitoes collected at different sentinel sites during mosquito activity season in 2018 in China**

| **Species** | ***Anopheles* spp.** | | ***Culex* spp.** | | ***Aedes albopictus*** | | ***Armigeres subalbatus*** | |
| --- | --- | --- | --- | --- | --- | --- | --- | --- |
| **Survey sites** | **No. Sample** | **No. pool** | **No. Sample** | **No. pool** | **No. Sample** | **No. pool** | **No. Sample** | **No. pool** |
| Zaoyang, Hubei | 273 | 28 | 2887 | 108 | 152 | 40 | 14 | 4 |
| Hohhot, Inner Mongolia |  |  | 22049 | 225 |  |  |  |  |
| Qionghai, Hainan | 115 | 12 | 1356 | 45 | 80 | 15 |  |  |
| Sanya, Hainan |  |  | 550 | 40 | 150 | 33 | 123 | 13 |
| Jining, Shandong | 94 | 4 | 363 | 36 | 129 | 9 |  |  |
| Jinshan, Shanghai | 4 | 3 | 536 | 100 | 42 | 21 |  |  |
| Jing’an, Shanghai |  |  | 208 | 69 | 160 | 65 |  |  |

**Table S2. Primer sets used for sequencing the GETV NM,JA_F2_18-8L-NH-Cxp-Y-1-1 strain**

| Primer | Position | Sequence, 5’-3^’^ | Polarity |
| --- | --- | --- | --- |
| 1-F | 1-19 | ATGGCGGACGTGTGACATC | Sense |
| 1-R | 1230-1250 | CTGAATGCTTGCGCTACCACT | Reverse |
| 2-F | 1084-1104 | ACCATATGCGACCAGATGACG | Sense |
| 2-R | 2221-2242 | GAACGCCAAACACACCAATAAT | Reverse |
| 3-F | 2046-2066 | AGTGAGAGCTGAAAGGGCAGA | Sense |
| 3-R | 3326-3344 | CGTCCTCCAGGCTTGTTGT | Reverse |
| 4-F | 3086-3108 | ATCCATTCCAAAACAAGGCTAAG | Sense |
| 4-R | 4347-4373 | TTGTTGGTACTAATTATGTTAGCCACA | Reverse |
| 5-F | 4184–4204 | TCGCTAAGAAGTGGCCATCAT | Sense |
| 5-R | 5442–5462 | GGAGGTGAGGTGCTAGTGGTT | Reverse |
| 6-F | 5225–5245 | AGCCGTACGTGACACAGCAAG | Sense |
| 6-R | 6418–6436 | CAGCAGCCTTTGGACCCTT | Reverse |
| 7-F | 6270–6295 | AATGAGAGAATTACCAATCATGGACT | Sense |
| 7-R | 7451–7473 | CTATTTAGGACCGCCGTACAGAT | Reverse |
| 8-F | 7302–7322 | TAAGTGGTCCCGCATAGGATT | Sense |
| 8-R | 8597–8617 | TCTATAGCCACCGGGCTGTAA | Reverse |
| 9-F | 8424–8446 | AGGATGTTAGAGGACAACGTGGA | Sense |
| 9-R | 9533–9553 | GGTTTGCCTTCAGTTGTCAGC | Reverse |
| 10-F | 9327–9347 | GCACCAGGTGTCACATACGGA | Sense |
| 10-R | 10500–10523 | CTGGTTGTAGACGTCGTTCTTGTA | Reverse |
| 11-F | 10289–10312 | TAGATCGGACGTATGCAAGCAC | Sense |
| 11-R | 11651–11678 | AAATATTAAAAAAACAAATTAGACGCCT | Reverse |

**Table S3. Primer sets used for sequencing the CHAOV NM,JA_F7_18-8L-NH-Cxp-Y-2-1 strain**

| Primer | Position | Sequence, 5’-3^’^ | Polarity |
| --- | --- | --- | --- |
| 1-F | 1–28 | GAAAAGTATATTCTACGTGTGCATTCTG | Sense |
| 1-R | 1217–1238 | GTGTCCTTTGCCTTGGTGTTAT | Reverse |
| 2-F | 1061–1083 | GGTGTGTGACTATTGCAGCAGAA | Sense |
| 2-R | 2255–2274 | GCTGCTGAAGAATCCTCCGA | Reverse |
| 3-F | 2058–2080 | GAAACAGAATGAGAAGGCAGTCG | Sense |
| 3-R | 3318–3342 | GTTTTCCACCACAACTTTAGTACCA | Reverse |
| 4-F | 3096–3115 | AGGCGACTGGAAACTGGAAT | Sense |
| 4-R | 4323–4344 | TGCGTAAGCCACAAAAATCAGA | Reverse |
| 5-F | 4159–4184 | GATCCATTAGGTATGCTTGGATACTC | Sense |
| 5-R | 5419–5442 | ATTAACCATTCTTCCTGGTGTCAA | Reverse |
| 6-F | 5251–5275 | AAGAAGTTGAAAACCCTTGTACTGG | Sense |
| 6-R | 6432–6454 | CAAATTCGATGAAAGCCTTGAGT | Reverse |
| 7-F | 6297–6316 | GTGCCATAGTGGGCCATCTG | Sense |
| 7-R | 7536–7560 | TGTGGAAGAGTTCCCTTCTATGAGT | Reverse |
| 8-F | 7352–7374 | AGAACCCTATTGTGGATGGAGTC | Sense |
| 8-R | 8626–8649 | TATCATTGATGAAGCACTTCCAGA | Reverse |
| 9-F | 8443–8465 | GAAGATGATGTCAACCTGGGAAC | Sense |
| 9-R | 9726–9747 | CATGCTGTTGAGAAAGTGCAGA | Reverse |
| 10-F | 9566–9590 | AAGGAGTGATCCTACCTGAAGACAC | Sense |
| 10-R | 10106–10127 | GCCAACATGTCTTCATTCGTCA | Reverse |
| 11-F | 9989–10011 | TCCACTTCCACAGAAGGGATCTA | Sense |
| 11-R | 10701–10725 | TTGTTTTACAACCTCCAAACTACCT | Reverse |

**Table S4. Primer sets used for sequencing the CxFV JS,JA_A4_18-9E-SJ-Cxp-Y-1-1 strain**

| Primer | Position | Sequence, 5’-3^’^ | Polarity |
| --- | --- | --- | --- |
| 1-F | 21–43 | TGGTTACACCGCAGATTGGTTA | Sense |
| 1-R | 1201–1220 | CACGATTGTAGGGCTGGGTT | Reverse |
| 2-F | 1070–1089 | ATATATGGCGGCCAATGGAT | Sense |
| 2-R | 2269–2289 | CTTTTCCAGACGAACCCTCCT | Reverse |
| 3-F | 2109–2129 | GCCTGTTGTCTAGCTCGACGA | Sense |
| 3-R | 3334–3357 | ATTGATGTCATCTCGTCCATGTCT | Reverse |
| 4-F | 3194–3214 | GCCAAGAAGTGGTGTTGCAAG | Sense |
| 4-R | 4413–4433 | CCGATACTCCAGCCACGTAGA | Reverse |
| 5-F | 4256–4278 | GATGGAGTGGAATTCACGAACTT | Sense |
| 5-R | 5501–5524 | TGTGGGAACGAATATTATCGTCTT | Reverse |
| 6-F | 5371–5391 | CGTGGTGTACATGAGTGCGAC | Sense |
| 6-R | 6615–6633 | AATCCCAACACCGCAACAA | Reverse |
| 7-F | 6436–6457 | TTGTAGTGGTTTGTCGGATCGT | Sense |
| 7-R | 7732–7753 | CACCAGACGTTGAGTCCATCCT | Reverse |
| 8-F | 7614–7635 | TGAACGAGACAGACAAGGGTGA | Sense |
| 8-R | 8894–8917 | CCACATGTACCAGATAATCCTTGA | Reverse |
| 9-F | 8663–8684 | CATGAACAACGTAAAAAGCGGA | Sense |
| 9-R | 9781–9799 | GCCGACGTGATAGCAGCAA | Reverse |
| 10-F | 9530–9552 | GTTCCCATCACTACCATCCTCTC | Sense |
| 10-R | 10131–10151 | GTCGTCAGTTGGAGCTCGGTA | Reverse |
| 11-F | 9983–10008 | AGGACATAAACTGTGGAAGTTTGATC | Sense |
| 11-R | 10780–10799 | GCCCGCAACAAGTCTCCTAA | Reverse |

**Table S5. Primer sets used for sequencing the QBV JS,JA_H6_18-10E-JS-Cxt-C-2-1 strain**

| Primer | Position | Sequence, 5’-3^’^ | Polarity |
| --- | --- | --- | --- |
| 1-F | 1–27 | GTCACTGGTTGATTAAGCCTATCTATT | Sense |
| 1-R | 1255–1278 | TACCCTTCTTCACAATGGAGTTCA | Reverse |
| 2-F | 1089–1107 | ACTGCCAGGTCAATGCCAC | Sense |
| 2-R | 2341–2362 | TGTGGTCTTCAGTTGTTCCGTT | Reverse |
| 3-F | 2176–2201 | TGGATACTTCTACTACACACGTGCTG | Sense |
| 3-R | 3405–3427 | AGGAGAAGATCCATCTACTGGCA | Reverse |
| 4-F | 3183–3203 | AGTGGTGCTGCAAAGGATGTT | Sense |
| 4-R | 5532–5551 | GGCAACGCCTTTCAAATCTC | Reverse |
| 5-F | 5322–5343 | TCATGGATTACCATAACGCGAA | Sense |
| 5-R | 6493–6514 | TCCACCTTGTGCATACGTGATT | Reverse |
| 6-F | 6303–6325 | TCATATCAAATGTGGACGACCCT | Sense |
| 6-R | 7542–7568 | CGTTCTTGTCAAGAGAATTCAATATCT | Reverse |
| 7-F | 7358–7384 | AACTATCTACTGGGCTTGCTAGACTAT | Sense |
| 7-R | 8551–8570 | GTGTGTCCGGCTCGACTGTT | Reverse |
| 8-F | 8341–8365 | GTTTGTTGATCGTGAACATCCTTAC | Sense |
| 8-R | 9495–9516 | GTTTTCGACGTGATCGTGATGT | Reverse |
| 9-F | 9299–9322 | TACGCTCTTAACACTGTGACCAAT | Sense |
| 9-R | 10394–10411 | CTTTCAGCGCCACGGGAT | Reverse |
| 10-F | 10138–10158 | AGTTCACCAGGATCCCGTGTT | Sense |
| 10-R | 10778–10796 | TGGAGATTTGCTGCTGCGA | Reverse |
